# Is miR-10a a tumor suppressor that modulates proliferation and invasion in high-grade bladder cancer?

**DOI:** 10.32604/or.2025.055306

**Published:** 2025-05-29

**Authors:** THAINá RODRIGUES, PATRíCIA CANDIDO, FERES CAMARGO MALUF, POLIANA ROMãO, CAROLINA MIE MIOSHI, VANESSA RIBEIRO GUIMARãES, JULIANA ALVES DE CAMARGO, KARINA SERAFIM DA SILVA, GABRIEL ARANTES DOS SANTOS, IRAN AMORIM SILVA, KATIA RAMOS MOREIRA LEITE, WILLIAM C. NAHAS, SABRINA T. REIS, RUAN PIMENTA, NAYARA IZABEL VIANA

**Affiliations:** 1 Laboratory of Medical Investigation (LIM55), Urology Department, Faculdade de Medicina da Universidade de São Paulo (FMUSP), São Paulo, 01246-903, Brazil; 2 Department of Biology, Instituto Federal de Educação, Ciência e Tecnologia de São Paulo (IFSP), Campus São Paulo, São Paulo, 01109-010, Brazil; 3 Moriah Institute of Science and Education (MISE), Hospital Moriah, São Paulo, 04084-002, Brazil; 4 Department of Biomedical Science, Centro Universitário São Camilo, São Paulo, 04263-200, Brazil; 5 Uro-Oncology Group, Urology Department, University of São Paulo Medical School and Institute of Cancer Estate of São Paulo (ICESP), São Paulo, 01246-000, Brazil; 6 Urology Department, D’Or Institute for Research and Education (ID’Or), São Paulo, 01401-002, Brazil; 7 Department of Bioscience, Universidade do Estado de Minas Gerais-UEMG, Passos, 37900-106, Brazil

**Keywords:** Bladder cancer (BC), MiR-10a, Tumor suppressor genes, Cell proliferation

## Abstract

**Objectives:**

Bladder Cancer (BC) is one of the most commonly diagnosed malignancies worldwide, with high rates of mortality and morbidity. It can be classified as non-muscle invasive bladder cancer (NMIBC) or muscle-invasive bladder cancer (MIBC), with radical cystectomy being the treatment for MIBC, which significantly reduces quality of life. MicroRNAs (miRs) act as critical genetic regulators, with both oncogenic and tumor-suppressive roles. MiR-10a is described as a tumor suppressor in various neoplasms, but its role in BC is controversial. This study aims to assess the activity of miR-10a in cellular invasion and proliferation in two distinct BC cell lines.

**Methods:**

The study used high-grade T24 and low-grade RT4 bladder cell lines. Cells were transfected with miR-10a mimic or a non-targeting control. Transfection efficiency was validated by qPCR. Cell proliferation was cultured for 10–14 days. Cell migration and invasion were evaluated using Matrigel. All assays were conducted in triplicate.

**Results:**

The T24 cells transfected with miR-10a presented decreased cellular proliferation and invasion compared to the Scramble (*p* = 0.0481 and *p* < 0.0001, respectively). In the RT4 cell line, there was only a significant reduction in cellular proliferation after miR-10a transfection (*p* = 0.0029). **Conclusions:** Our findings suggest that miR-10a has a tumoral suppressor role in BC, demonstrating higher efficacy in high-grade cells.

## Background

Bladder cancer (BC) is one of the most common carcinomas, associated with high rates of mortality and morbidity [1,2]. The most prevalent type, urothelial cancer (UC), is classified by the World Health Organization (WHO) into papillary urothelial neoplasia of low malignant potential (PUNLMP) [3,4], low-grade, and high-grade cancer [5,6]. Risk factors for BC include environmental and genetic elements that can lead to mutations affecting cellular proliferation, apoptosis, and differentiation [1,4].

Non-muscle invasive bladder cancer (NMIBC) is typically managed with transurethral resection of the bladder (TURB), followed by intravesical chemotherapy [4,5]. However, a significant portion of *in situ* tumors are high-risk, with increased rates of progression and metastasis [6,7]. Muscle-invasive bladder cancer (MIBC), is primarily treated with radical cystectomy and systemic chemotherapy, which carries substantial morbidity over the medium and long term [7,8].

Given these challenges, there is a growing interest in less invasive yet effective therapies for BC [9,10]. Advances in understanding epigenetics, DNA methylation, and microRNA (miRNA) have been pivotal. MiRNAs, small non-coding RNAs with post-transcriptional regulatory functions, are classified as oncogenic miRNAs or tumor-suppressor miRNAs, depending on their role in tumorigenesis [11–13].

MiR-10a has been identified as a tumor-suppressor miRNA in prostate cancer and as an oncomiRNA in cervical cancer [14,15]. However, its role in BC is more complex. Two studies by Dai et al. [16] and Li et al. [17] present conflicting results: the first found miR-10 underexpressed in BC samples and UC cell lines, while the second showed overexpression in stage I and II BC tissues, complicating our understanding of its function in BC [16,17]. These discrepancies may reflect phenotype-dependent expression of miR-10a, though the literature remains inconsistent, with studies reporting both under- and over-expression in different grades of BC [18,19].

This study aims to clarify miR-10a’s role by evaluating its activity in two distinct BC cell line phenotypes and determining whether its expression is stage-dependent in bladder tumorigenesis.

## Materials and Methods

### Cell culture and transfection

The *in vitro* models for BC were the T24 (high-grade BC) and RT4 (low-grade BC) cell lines, cultured according to the manufacturer’s guidelines (ATCC Culture Guides), before the experiments, the cells were tested for the presence of mycoplasma. Both T24 and RT4 cells were cultured in appropriate growth media (Roswell Park Memorial Institute (RPMI) 1640 Medium-RPMI-1640, 11875085, Thermo Fisher Scientific, Waltham, MA, USA), supplemented with 10% fetal bovine serum (FBS, Gibco™, A5670701, MA, USA) and 1% penicillin-streptomycin (15140122, Gibco). Cells were incubated at 37°C in a 5% CO₂ humidified incubator.

### MiR-10a cellular transfection

The lipofectamine transfection method was used to induce miR-10a expression. The procedure was conducted in triplicate with three conditions: the transfection complex alone, with the miR-10a *mimic* (MH10787), and with a *scramble/negative control* microRNA precursor (Ambion, Austin, TX, USA). The transfection complex was prepared by diluting the mimics in OPTI-MEM I medium (A4124801, Gibco). After dilution, the solution was mixed with the transfection reagent Lipofectamine RNAiMax (Invitrogen, 13778100, Carlsbad, CA, USA), following the manufacturer’s instructions. The final concentration of the transfection complex was 20 nM. The procedure was validated by quantitative reverse transcription polymerase chain reaction (qRT-PCR). The primer sequence used for the validation of miR-10a transfection was: UACCCUGUAGAUCCGAAUUUGUG.

### Genetic expression analysis—miRNA extraction and qPCR

miRNA was extracted from the cell lines using the mirVana kit (Ambion, AM1560, Austin, TX, USA) following the manufacturer’s instructions. The purity (260/280 nM ratio) and concentration of the genetic material were assessed using a Nanodrop® spectrophotometer (ND-1000, Wilmington, EUA), and the samples were stored at −80°C. Complementary DNA (cDNA) from miR-10a was synthesized using the TaqMan miRNA Reverse Transcription kit (Thermo Fisher, 4366596, MA, USA) as per the manufacturer’s instructions. The primer sequence used for the cDNA of miR-10a was: ACAAAUUCGGAUCUACAGGGUA. Target sequences were amplified using qPCR on the ABI 7500 Fast RT-PCR system (Applied Biosystems, 4351105, Waltham, MA, USA) with a reaction volume of 10 µL, containing 2 µL of HOT FIREPol Probe Universal qPCR mix (Solis BioDyne, 08-88-0000S, Tartu, Estonia) and 0,5 µL of a specific probe. All reactions were performed in duplicate, using RNU48 as an endogenous control (Sequence: AGTGATGATG ACCCCAGGTA ACTCTGAGTG TGTCGCTGAT GCCATCACCG CAGCGCTCTGACC).

Expression levels were calculated using the relative quantification method. Data analysis was conducted with DataAssist software version: 3.01 (Applied Biosystems, USA).

### Colony formation assay

Cells were seeded at low density (3 × 10^2^ cells/well) in 12-well plates and transfected after 48 h. They were then incubated at 37°C with 5% CO_2_ for 10 days. Colonies were fixed (using 4% paraformaldehyde), stained using 1 mL of 3% methylene blue (Millipore Sigma, M4159, Maryland) for 30 min (2×), washed with PBS, and counted. Colonies smaller than 1 mm were excluded. Images were captured and analyzed with ImageJ software (National Institutes of Health, Bethesda, MD, USA, Version 1.54j).

### Cell invasion assay

Following transfection, T24, and RT4 cells were seeded at an inoculation density of 1 × 10^5^ cells per well into separate Corning BioCoat Matrigel Invasion chambers (Becton Dickinson, Bedford, MA, USA), containing 24 wells with 8 µm pores coated with Matrigel (BD Biosciences, 356255, San Jose, CA, EUA). Matrigel was diluted in 450 µL of serum-free medium, seeded with 1 × 10^4^ cells/mL in 250 µL of serum-free medium, and 750 µL of medium supplemented with 10% FBS was added to the lower chambers. The cells were incubated for 48 h at 37°C with CO_2_ at 5%, then fixed with 4% formaldehyde in PBS, stained with 0.2% crystal violet in methanol, and counted under an optical microscope (Nikon Model Eclipse E200, Tokyo, Japan) at 200× magnification.

### Statistical analysis

Descriptive analysis was presented as mean and standard deviation (SD) Student’s *t*-test (for parametric variables) or Mann-Whitney test (for nonparametric variables) was used for group comparisons. All assays were repeated three times (n = 3) to ensure the reliability of the results. Analyses were performed using GraphPad Prism 10 (GraphPad Software Inc., San Diego, CA, USA). A *p*-value < 0.05 was considered statistically significant, with a 5% significance level applied for all tests.

## Results

### Overexpression of miR-10a in the T24 cell lines

**Figure 1:**
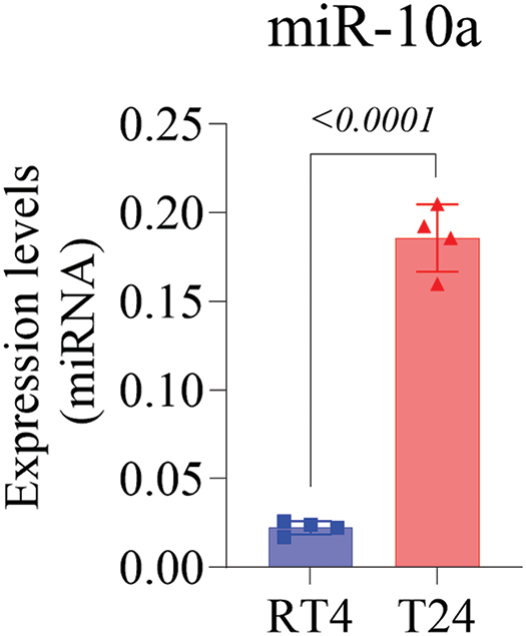
MiR-10a expression levels in non-manipulated T24 and RT4 cell lines, showing higher expression in T24 (*p* < 0.0001). Data represent mean ± SD from three independent experiments (n = 3).

Genetic expression analysis showed that the T24 cell line had significantly higher miR-10a expression than the RT4 cell line (*p* < 0.0001; Fig. 1).

### Validation of MiR-10a transfection in BC cell lines

**Figure 2:**
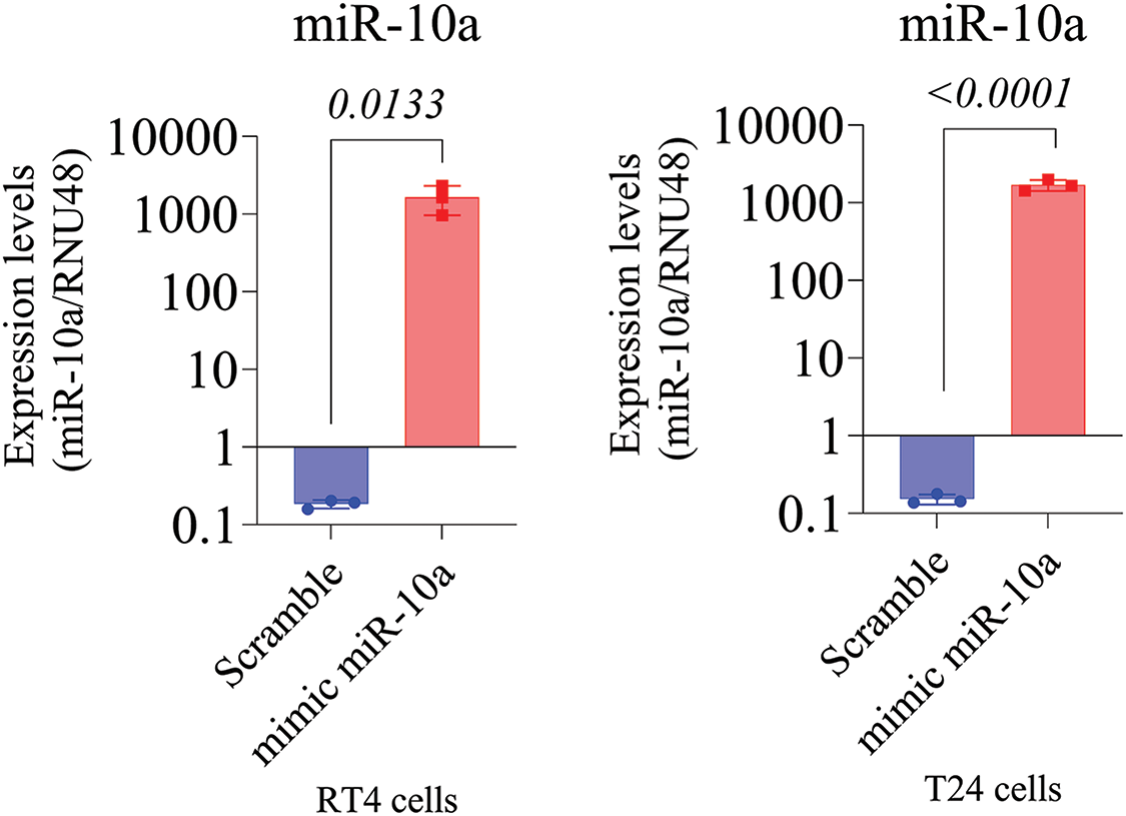
MiR-10a expression in transfected T24 and RT4 cells. A) MiR-10a expression in transfected T24 cells showing a 1000-fold compared to control (*p* = 0.0133). B) MiR-10a expression level in transfected RT4 cells, showing a 1000-fold increase compared to control (*p* < 0.0001). Data represent mean ± SD from three independent experiments (n = 3).

miR-10a transfection was validated in the T24 and RT4 cell lines via q-PCR, demonstrating a 1000-fold increase in miR-10a concentration in transfected groups compared to scramble controls (*p* = 0.0133 for T24, *p* < 0.0001 for RT4; Fig. 2A and B).

### Inhibitory effect of miR-10a on cell proliferation in BC cell lines

**Figure 3:**
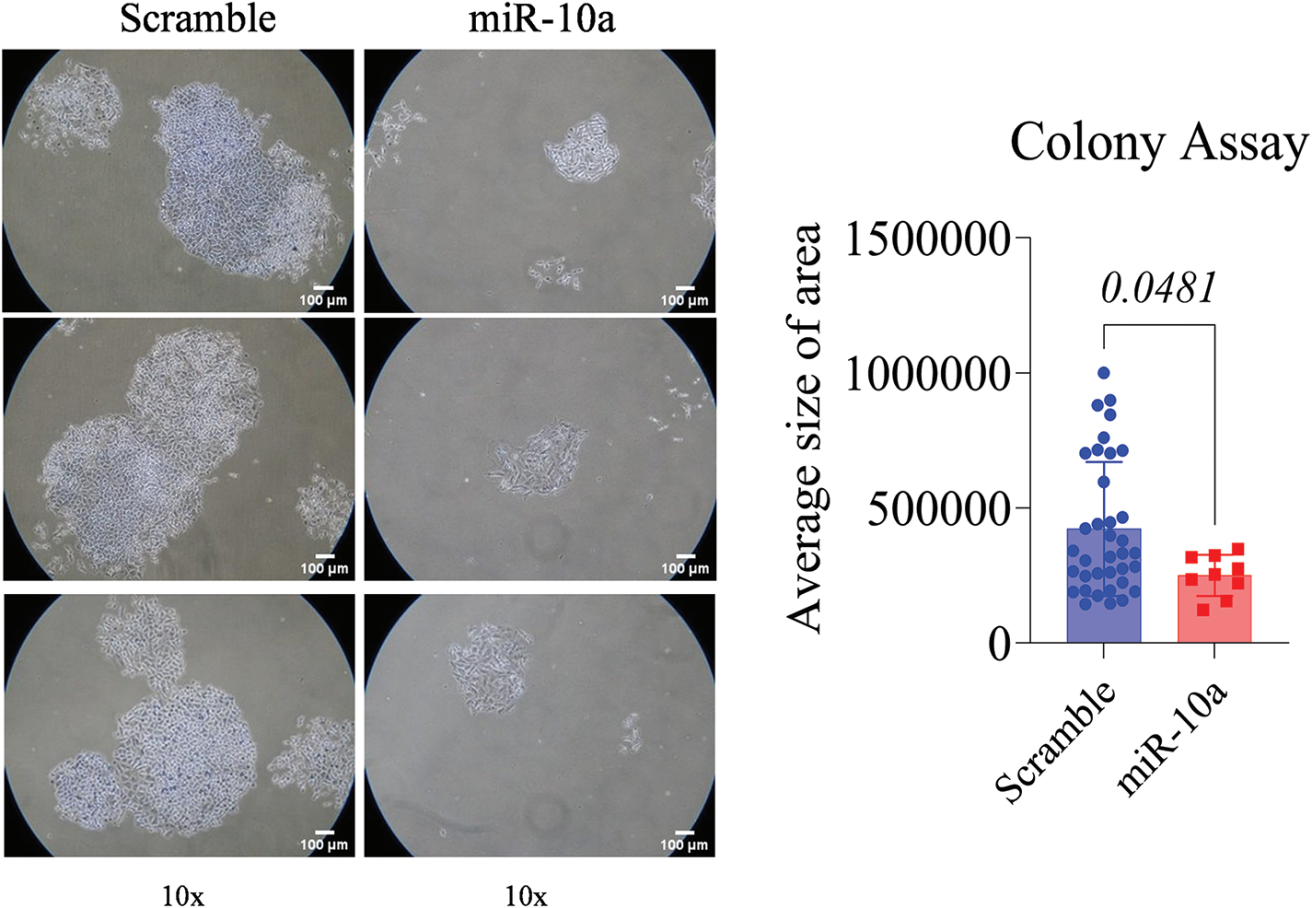
Colony formation assay in transfected T24 cells, showing s reduced colony area compared to control (*p* = 0.0481). Data represent mean ± SD from three independent experiments (n = 3).

**Figure 4:**
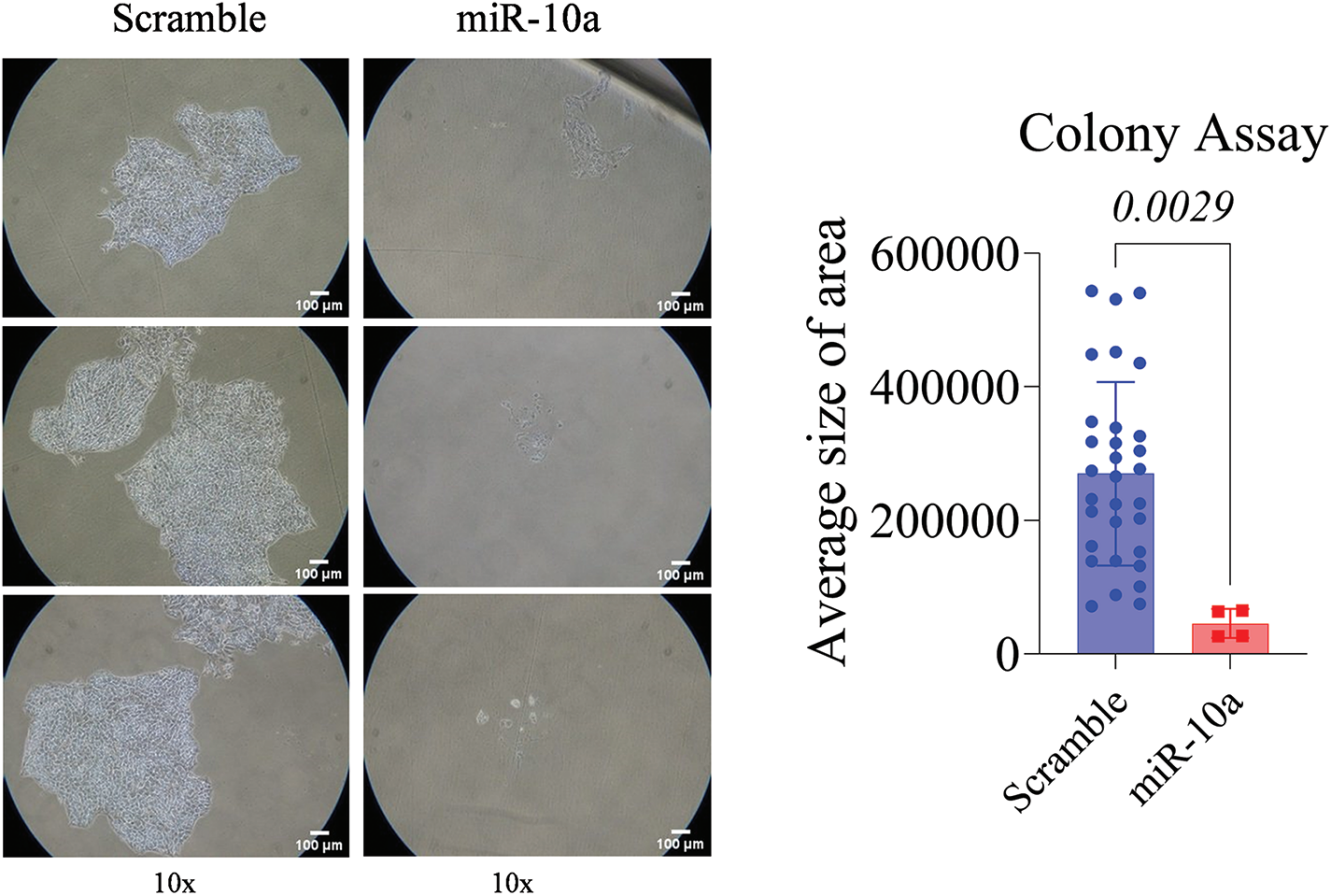
Colony formation assay in transfected RT4 cells, showing reduced colony area compared to control (*p* = 0.0029). Data represent mean ± SD from three independent experiments (n = 3).

A significant reduction in proliferation was observed in the miR-10a transfected groups compared to the scramble controls, with *p* = 0.0481 for T24 and *p* = 0.0029 for RT4 (Figs. 3 and 4).

### Invasion assay: inhibitory effect of miR-10a in T24 cells

**Figure 5:**
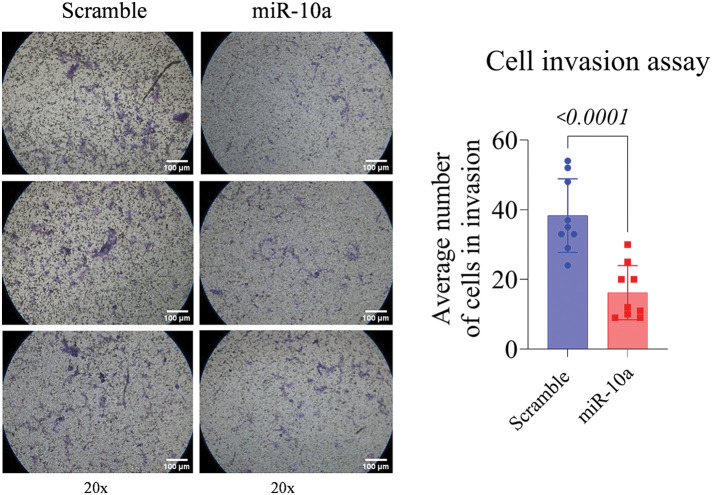
Invasion assay in transfected T24 cells, showing reduced invasion rate compared to control (*p* < 0.0001). Data represent mean ± SD from three independent experiments (n = 3).

**Figure 6:**
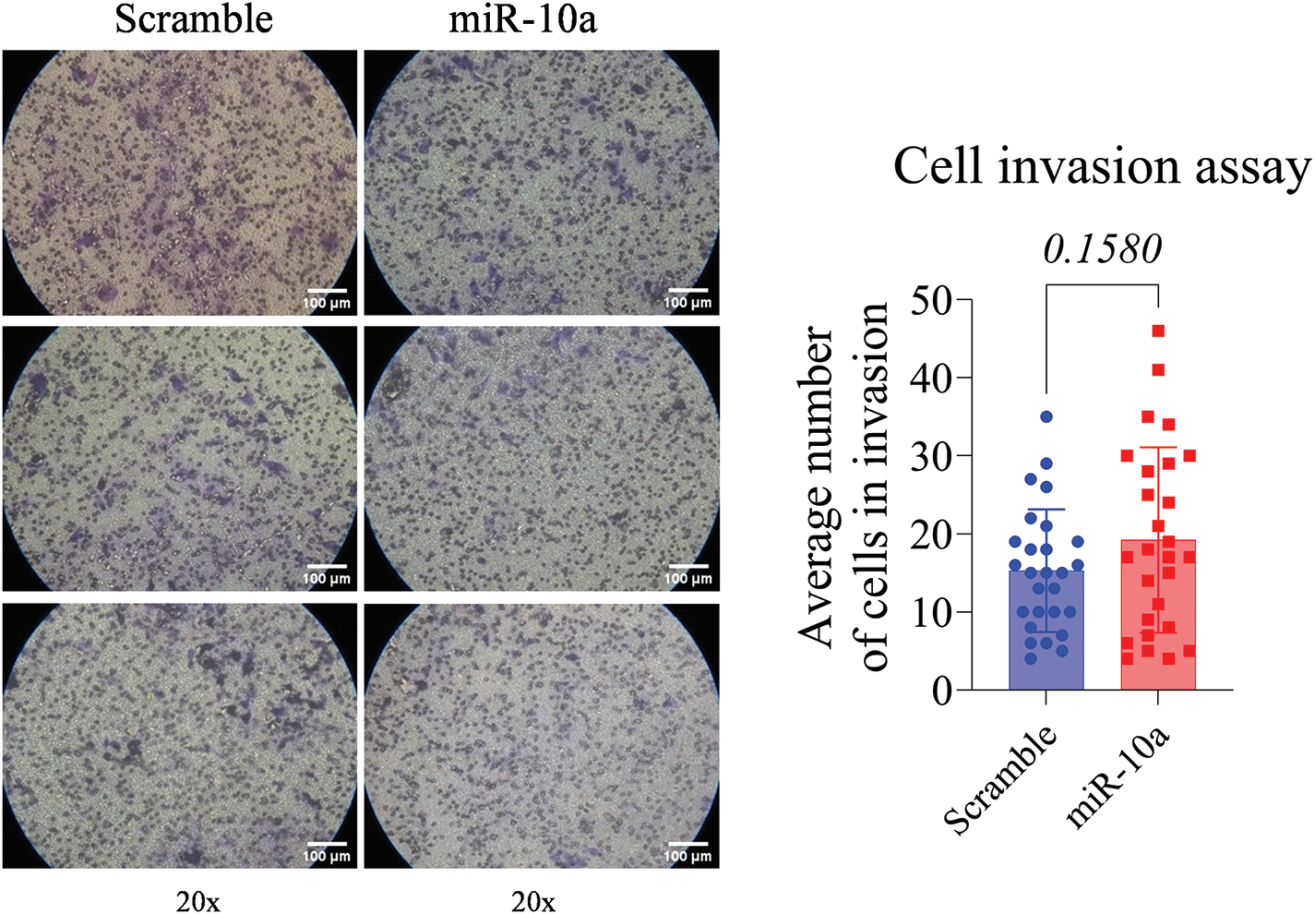
Invasion assay in transfected RT4 cells, showing no significant difference between groups (*p* = 0.1580). Data represent mean ± SD from three independent experiments (n = 3).

The T24 cells showed a reduced invasion rate compared to the control (*p* < 0.0001). No significant difference was detected between e groups in the RT4 cell line (*p* = 0.158, Figs. 5 and 6).

## Discussion

In recent years, miRNAs have been recognized as significant regulators of cell development and tumorigenesis, with applications in diagnosing and differentiating disease stages [20]. Moreover, many studies have demonstrated their potential in treating malignancies, particularly challenging ones like high-grade BC [21,22]. Our data contribute to understanding the inhibitory role of miR-10a in BC, regardless of disease stage.

Initial analysis of miR-10a concentration in non-transfected cell lines revealed increased expression in high-grade disease, while low-grade BC was associated with decreased expression, consistent with findings by Yang et al. [23]. Their study showed under-expression of miR-10a in grade I BC cells compared to normal urothelial cells. For high-grade diseases, Köhler and colleagues categorized them into grades II and III, as per the 1973 WHO classification. MiR-10a was upregulated in grade II but downregulated in grade III BC cells. Similarly, Dip et al. [18] found miR-10a under-expression in high-grade pT2-T3 samples compared to benign ones, with overexpression in high-grade samples linked to poorer prognosis [18]. These findings suggest stage-dependent expression, possibly due to DNA methylation differences affecting transcription [19]. Epigenetic modifications, including promoter hypermethylation, have been reported as key regulators of miRNA expression in BCa [24]. Our data reinforce this phenotype-dependent expression of miR-10a, despite the lack of consensus in the literature.

In the colony formation assay, miR-10a transfection showed a negative effect on cellular proliferation in both cell lines. This tumor-suppressor role in BC has been described by Dai et al. [16], where miR-10a upregulation reduced proliferation in two urothelial cancer cell lines (EJ and 253J), consistent with our findings. However, Li et al. [17] did not associate miR-10 with proliferation in two other cell lines (HT-1197 and HT-1376) using a CCK-8 assay. Notably, both studies assessed miR-10a expression secondary to another regulator, rather than as a primary intervention. Our findings support the inhibitory effect of miR-10a on BC cell proliferation.

Regarding the invasion assay, miR-10a’s suppressor effect was observed only in the high-grade cell line. In contrast, Dip et al. [18] found a positive association between miR-10 induction and cell invasion in other high-grade cell lines (HT-1197 and HT-1376). Xiao et al. [25] using a miR-10b mimic in BC cell lines and nude mice, reported an increase in miR-10a expression in BC cell lines, especially high-grade J82 cells, aligning with our findings. However, their transfection experiments indicated that miR-10b enhances metastatic potential by targeting tumor-suppressor genes KLF4 and HOXD10 [25].

Our results differ from those of Yang et al. [23], who found that miR-10a-5p mimics upregulation in high-grade BC cell lines in increased invasion, proliferation, and migration of cells. Two factors may explain these discrepancies: genomic variability between the cell lines, even among those classified as high-grade UC, and the use of different miR-10 family members across studies [23]. Although similar, the miR-10a and miR-10b may have distinct functions, leading to divergent results [23,26,27].

miR-10a did not significantly affect the invasion potential in the RT4 cell line but did in the T24 cell line. We hypothesize that the heterogeneity in proliferation rates, among low-grade suggests that miR-10a is more effective in cells with higher division rates. Differences in cellular metabolism could also explain the variation between cell lines [26]. Zhao et al. [27] found that T24 cells, which had a faster metabolism and higher division rate than RT4 cells, might be more influenced by miR-10a.

One limitation of our study is the difficulty in comparing findings across the literature due to the use of different BC cell lines and miR-10 family members. A recent systematic review identified 157 human BC cell lines, each with unique gene mutations, locations, and clinicopathological characteristics [28]. Previous studies have also emphasized the importance of selecting appropriate BC cell models to ensure translational relevance of *in vitro* findings [29]. Future studies should aim to define the most appropriate BC cell line and miR-10 member for investigation.

Our results suggest that miR-10a may act as an inhibitor of BC tumorigenesis, particularly in malignancy proliferation and cellular invasion, which are critical to metastasis and tumor progression. However, further studies are necessary to clarify the role of miR-10a across different stages of BC and its potential as a molecular-targeted therapy, as suggested by Singh et al., who explored the dual roles of miR-10a as a tumor suppressor and its therapeutic potential in various cancers [30].

## Conclusions

This study revealed promising evidence regarding the regulation of miR-10a in BC, highlighting its inhibitory capacity in cellular invasion and progression. The molecule was found to be more effective in high-grade cells, indicating its increasing importance in the advanced stages of BC. These findings suggest the therapeutic potential of miR-10a in the treatment of BC. However, further research is needed to better understand the underlying mechanisms of miR-10 activity and its clinical applications.

## Data Availability

The data that support the findings of this study are available from the corresponding author upon reasonable request.
